# Supplementary material for: TrkB dependent adult hippocampal progenitor differentiation mediates sustained ketamine antidepressant response
Source: Nat Commun. 2017 Nov 21;8:1668. doi: 10.1038/s41467-017-01709-8 (PMC5698402; doi:10.1038/s41467-017-01709-8)
Supplement: Supplementary file 1 — Supplementary Information [file 41467_2017_1709_MOESM1_ESM.pdf]

Supplementary Fig. 1

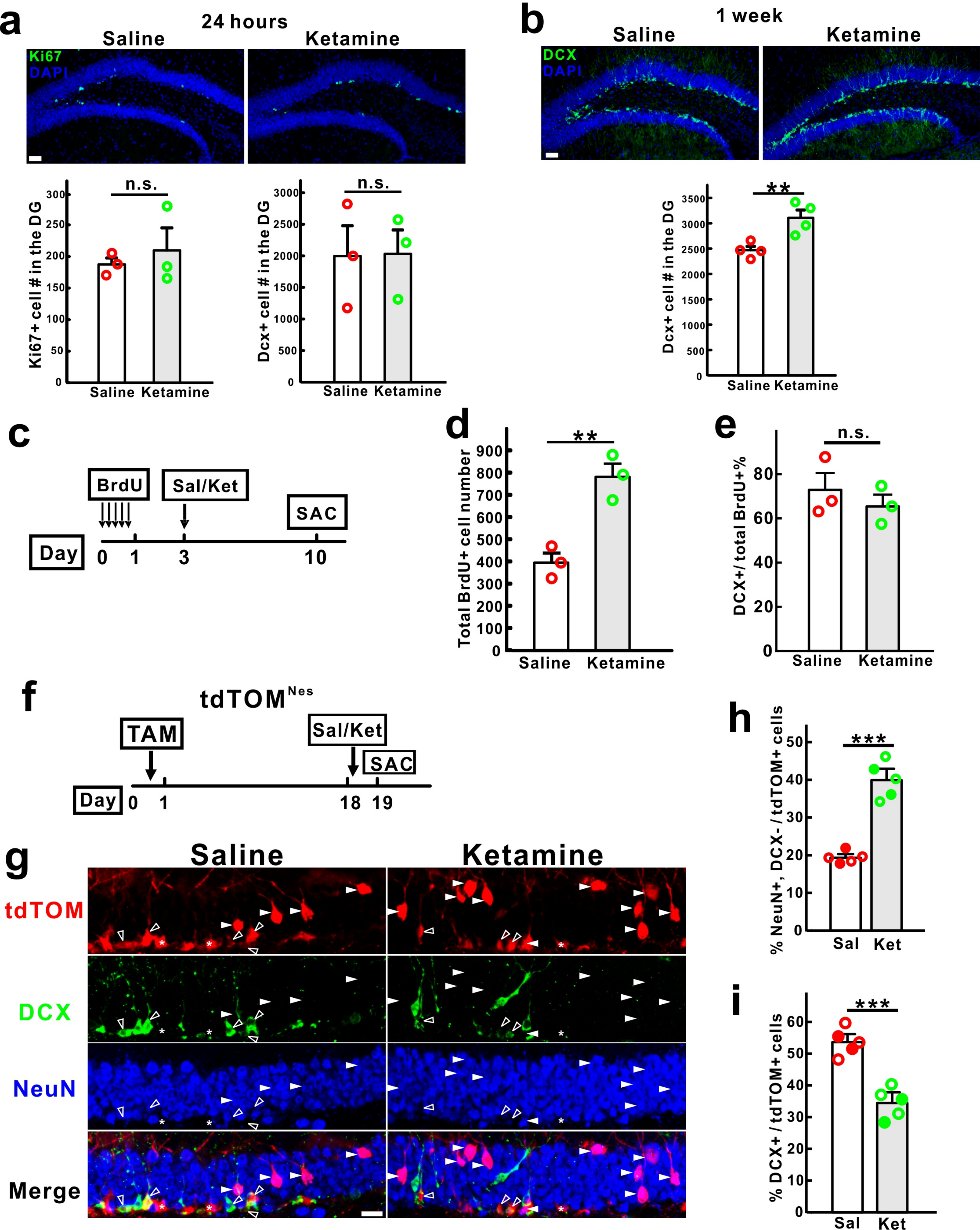

Supplementary Figure 1. Ketamine has no effect on early neural progenitor proliferation and differentiation. (**a**) Representative images of immunostaining with Ki67 to assess NPC proliferation in the adult DG 24 hours after ketamine treatment (upper panel). Histogram (lower panel) shows the quantification of relative Ki67 and DCX positive cell number in the DG (n = 3 per group; p = 0.5816 for Ki67 and p = 0.9601 for DCX by unpaired two-tailed *t*-test).

(**b**) Representative images of DCX staining 1 week after treatment (upper panel). Quantitation of relative DCX positive cell number in DG (lower panel, n = 4 per group; **p = 0.0096 by unpaired two-tailed *t*-test).

(**c, d, e**) Diagram for measuring NPC differentiation is shown in (c). Mice were treated with saline or ketamine 3 days after BrdU pulses, and 7 days later, mice were sacrificed to measure total BrdU+ cell number (d) and the percentage of BrdU+ cells that express DCX (e) in the DG (n = 3 per group; **p = 0.005 for BrdU+ cell number; p = 0.8346 for the percentage; unpaired two-tailed *t*-test).

(**f**) Nestin-creERt2; tdTOM ($tdTOM^{Nes}$) mice were induced with one dose of tamoxifen 18 days before saline (Sal) or ketamine (Ket) treatment, and animals were sacrificed (SAC) at 24 hours after the treatment.

(**g**) Representative confocal images of the DG show tdTOM+ cells (red) that co-express DCX (green, denoted by empty arrowheads) or NeuN alone (blue, denoted by solid arrowheads). Some of the tdTOM+ cells (denoted by stars) are not colocalized with NeuN and DCX, indicating they are maintained in the neural stem cell pool. Scale bar, 20 µm.

(**h**) Quantification of tdTOM+/NeuN+/DCX- cell percentage in total tdTOM+ cells after 24 hours of the treatment (n = 5 for each condition; unpaired two-tailed *t*-test, ***p < 0.0001).

(**i**) Quantification of tdTOM+/DCX+ cell percentage in total tdTOM+ cells after 24 hours of the treatment (n = 5 for each condition; unpaired two-tailed *t*-test, ***p = 0.0003). Circles in solid color denote the data points from female mice.

Supplementary Figure 2

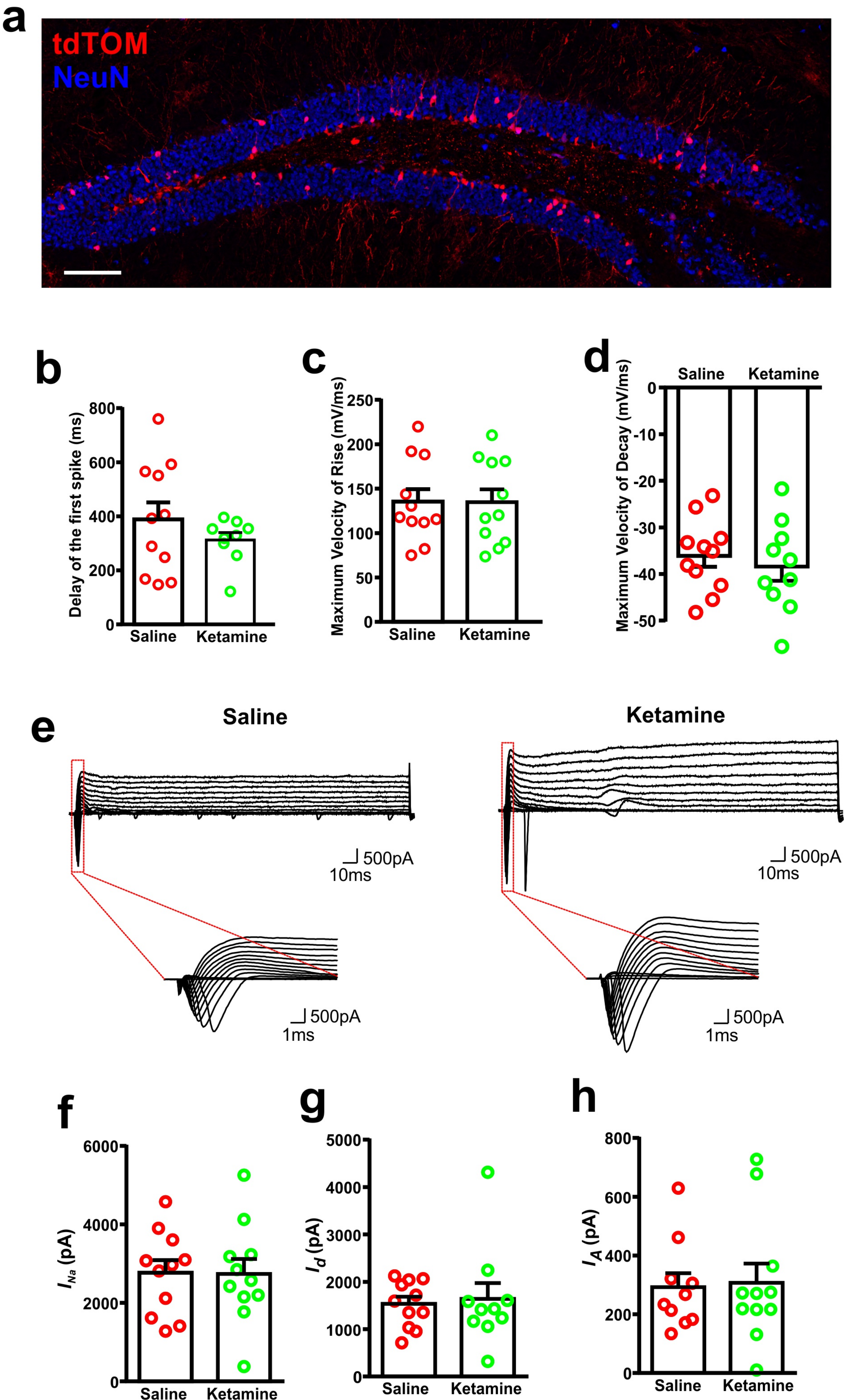

Supplementary Figure 2. Additional electrophysiology properties of newborn neurons.

(**a**) A representative thin section shows that Nestin-creER^{T2} efficiently recombines the Rosa26-tdTOM transgene in the neural stem/progenitor cells and newborn neurons in the dentate gyrus three weeks after the tamoxifen induction. Scale bar, 100 µm.

(**b**) A plot of neuron's delay of the first spike under saline or ketamine (saline, n=11; ketamine, n=9; p = 0.3131; not significant by unpaired two-tailed *t*-test).

(**c**) A plot of neuron's maximum velocity of rising under saline or ketamine (n=11 per group; p = 0.9717; not significant by unpaired two-tailed *t*-test).

(**d**) A plot of neuron's maximum velocity of decay under saline or ketamine (saline, n=11; ketamine, n=10; p = 0.5581; not significant by unpaired two-tailed *t*-test).

(**e**) Representative waveforms of inward and outward currents triggered by voltage steps, indicating sodium and potassium currents, respectively.

(**f**) A plot of neuron's approximate sodium current (I_{Na}) under saline or ketamine (n=11 per group; p = 0.9506; not significant by unpaired two-tailed *t*-test).

(**g**) A plot of neuron's approximate delayed potassium current (I_d) under saline or ketamine (saline, n=11; ketamine, n=10; p = 0.8633; not significant by Mann-Whitney test).

(**h**) A plot of differentiated neuron's approximate A-type potassium current (I_A) under saline or ketamine (saline, n=10; ketamine, n=11; p = 0.8633; not significant by Mann-Whitney test).

Supplementary Fig.3

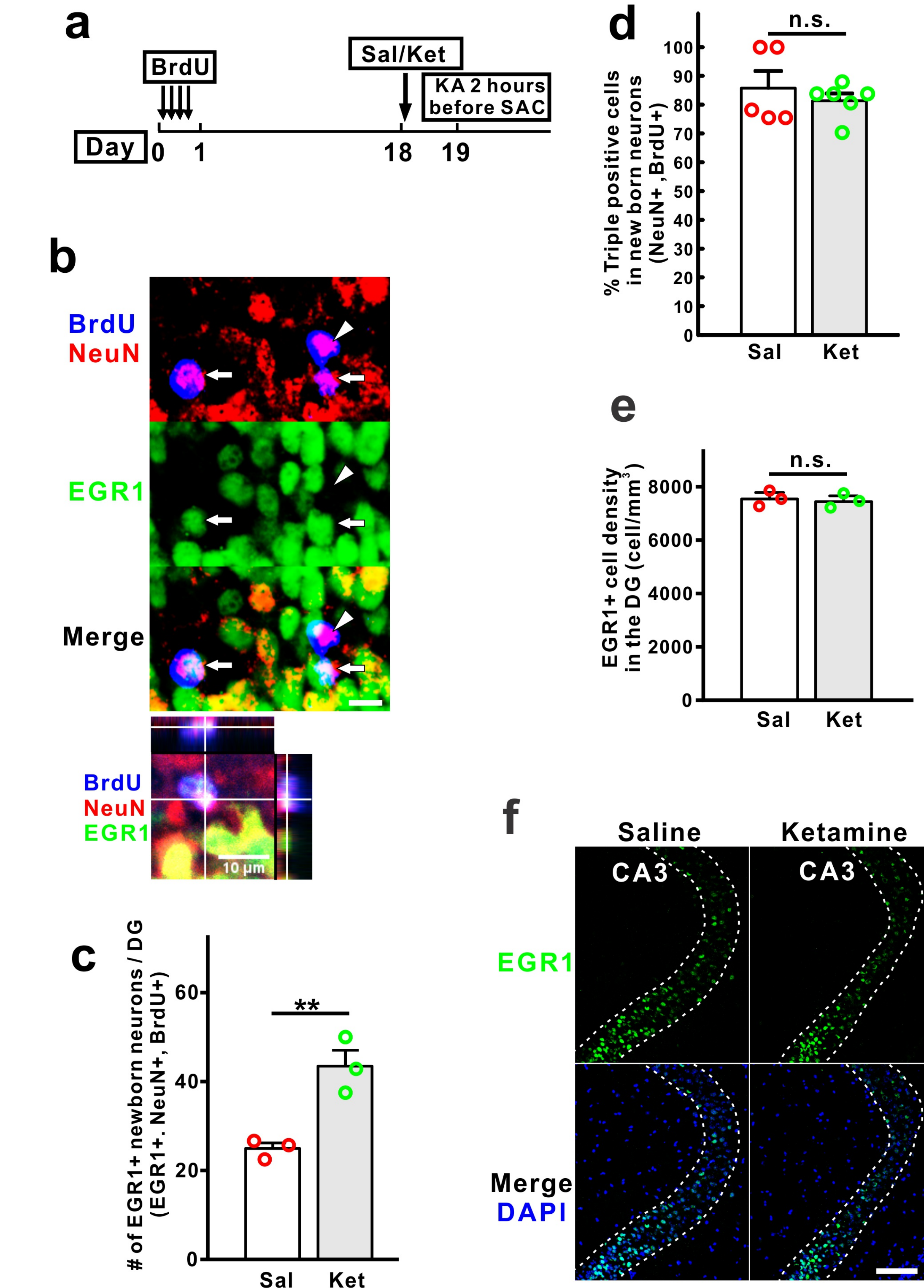

Supplementary Figure 3. Ketamine-induced newborn neurons exhibit EGR1 activation after kainate stimulation.

(**a**) Eighteen days after BrdU pulses, mice were treated with saline (Sal) or ketamine (Ket) for 24 hours and injected with KA (35 mg/kg, i.p.) two hours before sacrifice.

(**b**) Representative confocal images in the upper panel show newborn neurons that are stained with BrdU (blue) and NeuN (red) (highlighted by both arrows and arrowhead). In the middle panel, cells denoted by arrows co-express EGR1 (green), while the cell highlighted by arrowhead do not have EGR1 expression. Image with an orthogonal view of a triple positive cell is presented on the right. Scale bar, 10 µm.

(**c**) Quantification of EGR1 positive newborn neurons (BrdU+/NeuN+/EGR1+) in the DG after saline (Sal) or ketamine (Ket) treatment (n = 3 per group; unpaired two-tailed *t*-test, **p = 0.0085).

(**d**) The percentage of newborn neurons (BrdU+/NeuN+) that have EGR1 co-expression after saline (Sal) or ketamine (Ket) treatment (n = 5, 6 for saline and ketamine group, respectively; p = 0.4859, not significant (n.s.) by unpaired two-tailed *t*-test).

(**e**) The density of EGR1+ neurons in the DG was measured in both saline (sal) and ketamine (ket) treated mice (n = 3 per group; p = 0.9085, not significant (n.s.) by unpaired two-tailed *t*-test).

(**f**) Representative images of EGR1 immunofluorescence in the CA3 of saline and ketamine-treated mice show no difference in the neuron activity. Scale bar, 100 µm.

Supplementary Fig.4

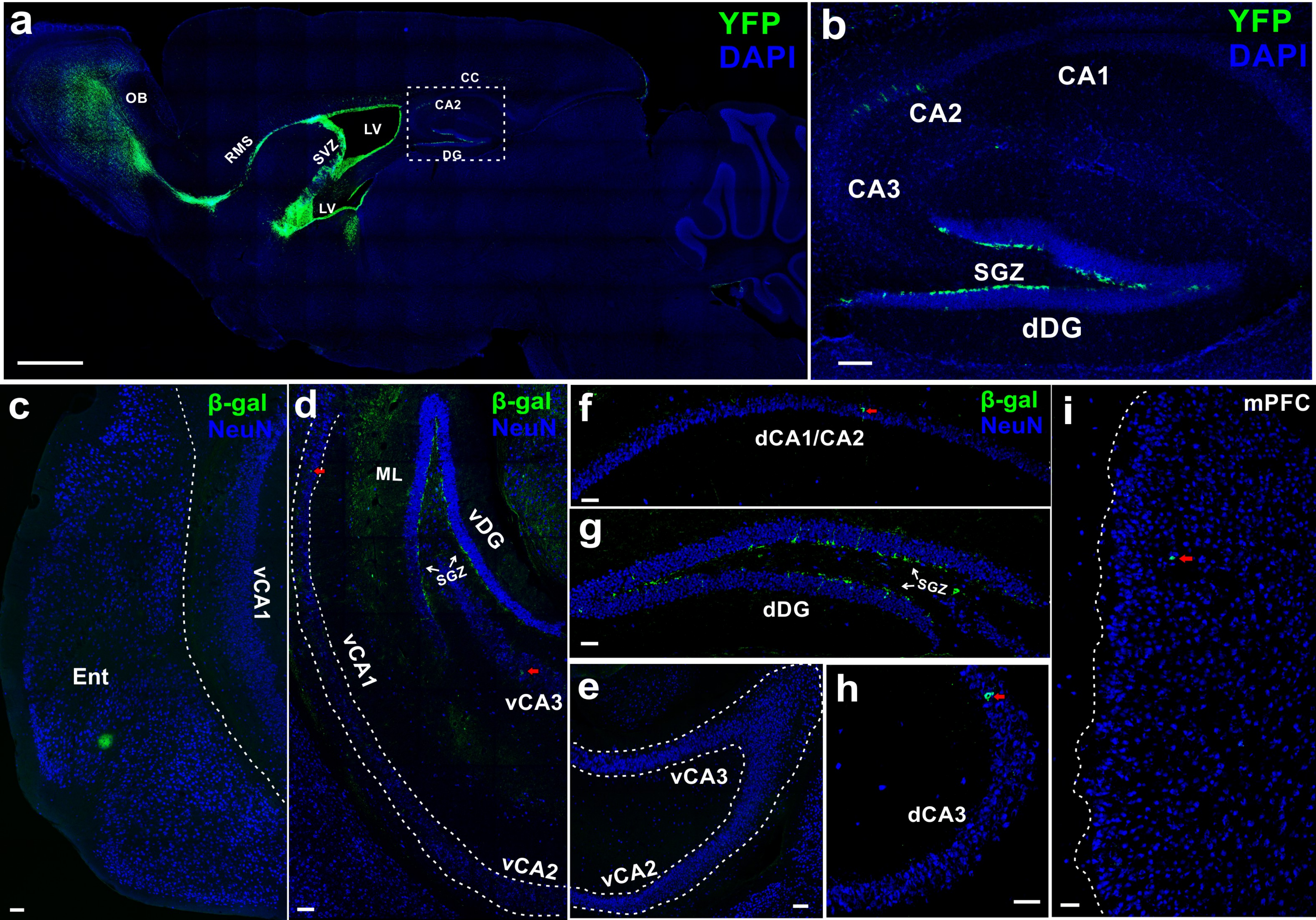

Supplementary Figure 4. Nestin-creER^{T2} transgene expression is restricted to the neurogenic niche.

(**a**) A sagittal overview of Nestin-creER^{T2}; Rosa26-YFP adult mouse brain two weeks after tamoxifen induction. YFP+ adult stem/progenitor cells (green) are highly concentrated in the subventricular zone (SVZ) of the lateral ventricle (LV) and then differentiate to their progenies, which migrate along the rostral migratory stream (RMS) to the olfactory bulb (OB). YFP+ stem/progenitor cells are also present in the subgranular zone (SGZ) of the dentate gyrus (DG). Very few YFP+ cells are spotted in some other regions, the CA2 region of the hippocampus and corpus callosum (CC). Scale bar, 1000 µm. (**b**) The magnified image for the hippocampus area in the dash-line rectangle is also shown. Scale bar, 100 µm.

(**c-i**) β-galactosidase (β-gal) staining was performed on coronal sections of both ventral (v) and dorsal (d) hippocampus including entorhinal cotex (Ent), vCA1/CA2/CA3, vDG & molecular layer (ML) (c, d, e), dCA1/CA2 (f), dDG (g), dCA3 (h), and mPFC (i) in Nestin-creER^{T2}; Rosa26-LacZ mice. All representative images that are from the same animal, clearly show that β-gal positive cells (green) are restricted to the subgranular zone (SGZ) of the DG three weeks after tamoxifen induction. Some β-gal labeled newborn cells become NeuN positive neurons (blue) in the granular layer (GL) of both dorsal and ventral DG (d, g), but are rarely detected in CA1, CA3 and mPFC (red arrows, in d, f, h, i). Scale bar, (c, d, e) 100 µm; (f, g, h, i) 50 µm.

Supplementary Fig.5

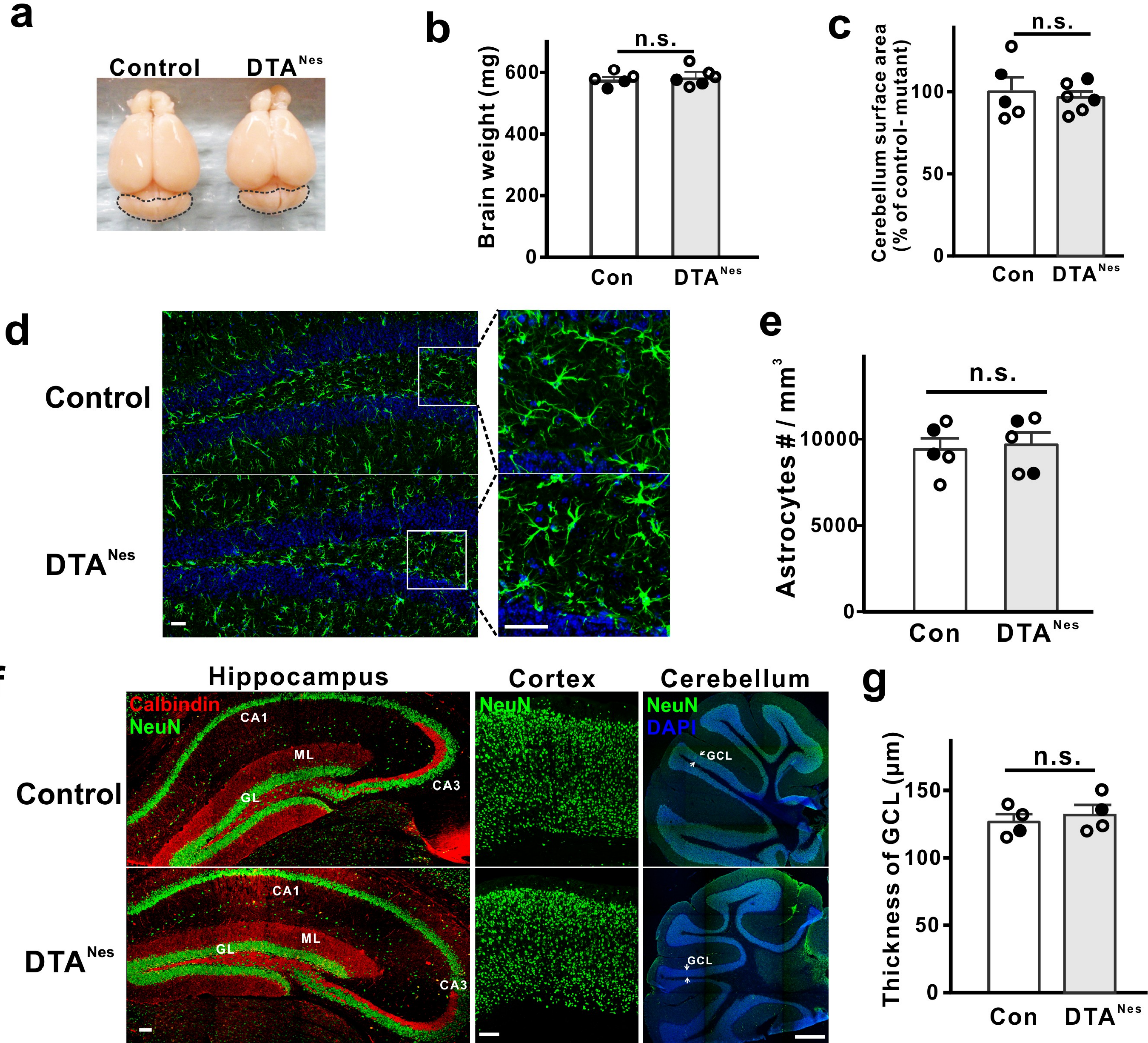

Supplementary Figure 5. DTA-mediated cell death does not affect granule neurons and brain morphology. (**a**) No obvious brain morphological abnormalities in DTA^{Nes} mice compared to littermate controls. The cerebellum has been circled by the black dashed lines.

(**b**) Brain weight is not significantly changed in DTA^{Nes} mice (n= 5, 6 for each group, respectively, p = 0.6207, not significant (n.s.) by unpaired two-tailed *t*-test).

(**c**) The cerebellum surface area quantified with pixel area in the control and mutant is shown (n= 5, 6 for each group, respectively, p = 0.6866, not significant (n.s.) by unpaired two-tailed *t*-test).

(**d**) Immunostaining of the DG with GFAP (green) for mature astrocytes shows no obvious astrogliosis and morphology change. Magnified images of the astrocytes in the hilus region are also shown for each condition. Sections counter-stained with DAPI (blue) display similar thickness of the granular layer. Scale bar, 20 µm.

(**e**) Quantification of mature astrocytes in the DG shows no difference in cell density between the control and DTA^{Nes} mice (n= 5, 5 for each group, respectively, p = 0.7802, not significant (n.s.) by unpaired two-tailed *t*-test).

(**f, g**) Immunostaining of the hippocampus, frontal cortex and cerebellum with calbindin (red) and/or NeuN (green) shows that DTA transgene expression driven by Nestin-$creER^{T2}$ has no effect on the mature neuron survival and brain region structure in the hippocampus, prefrontal cortex, and cerebellum. CA1, CA3, GL (granular layer) and ML (molecular layer) areas are labeled in the hippocampus. A representative area of granule cell layer (GCL) in the cerebellum is marked by two arrows, and the thickness of GCL was quantified and shown in (g) (n= 4, 4 for each group, respectively, p = 0.5981, not significant (n.s.) by unpaired two-tailed *t*-test). Scale bar, 100 µm for the images of hippocampus and cortex; 500 µm for the cerebellum images.

Circles in solid color denote the data points from female mice.

Supplementary Fig. 6

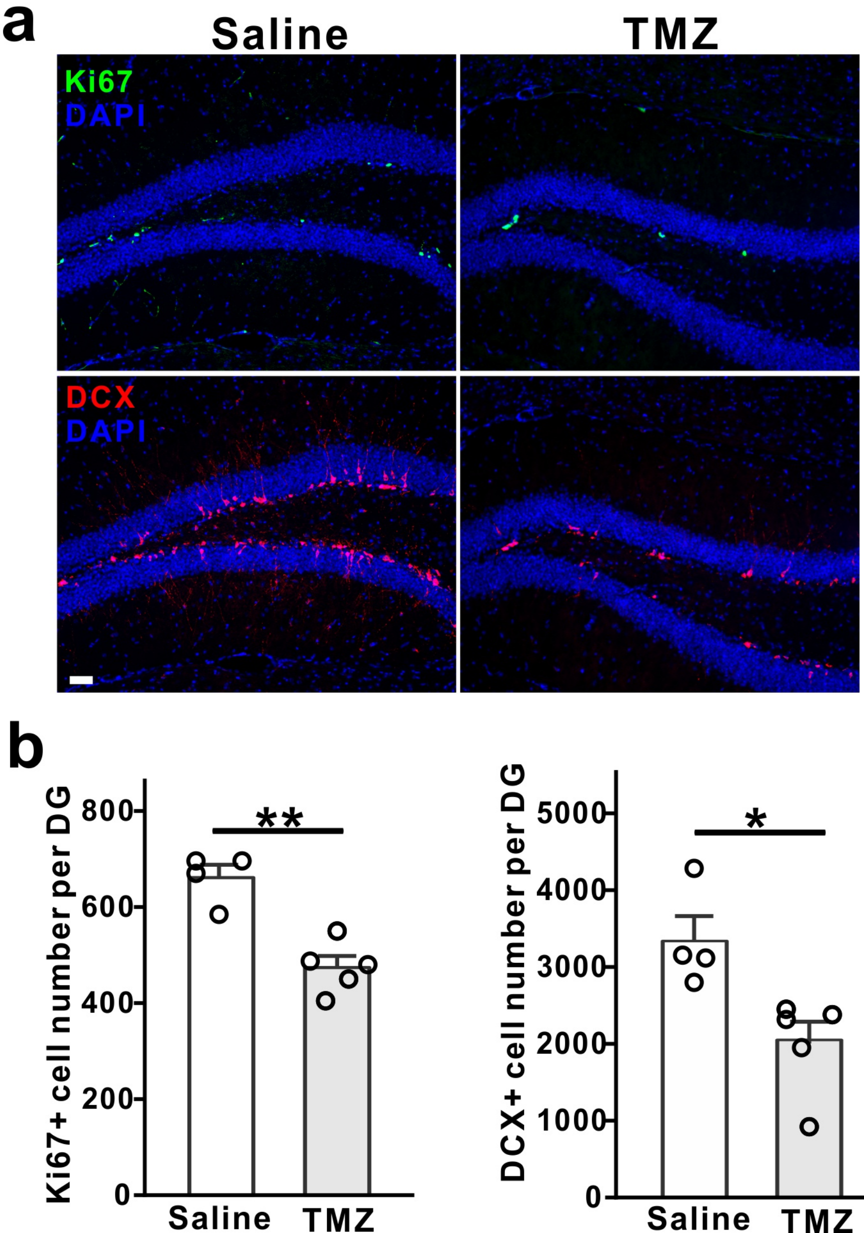

Supplementary Figure 6. TMZ treatment efficiently reduces NPC proliferation and differentiation.

(**a**) Representative images of Ki67+ (green) and DCX+ (red) cells in the DG after four cycles of TMZ treatment. Sections were counterstained with DAPI (blue) for nuclei. Scale bar, 50 µm.

(**b**) The quantification shows that both Ki67 and DCX cell numbers are decreased by the TMZ treatment (n = 4, 5 for saline and TMZ group respectively, **p = 0.0012 for Ki67, *p = 0.0202 for DCX by unpaired two-tailed *t*-test).

Supplementary Figure 7

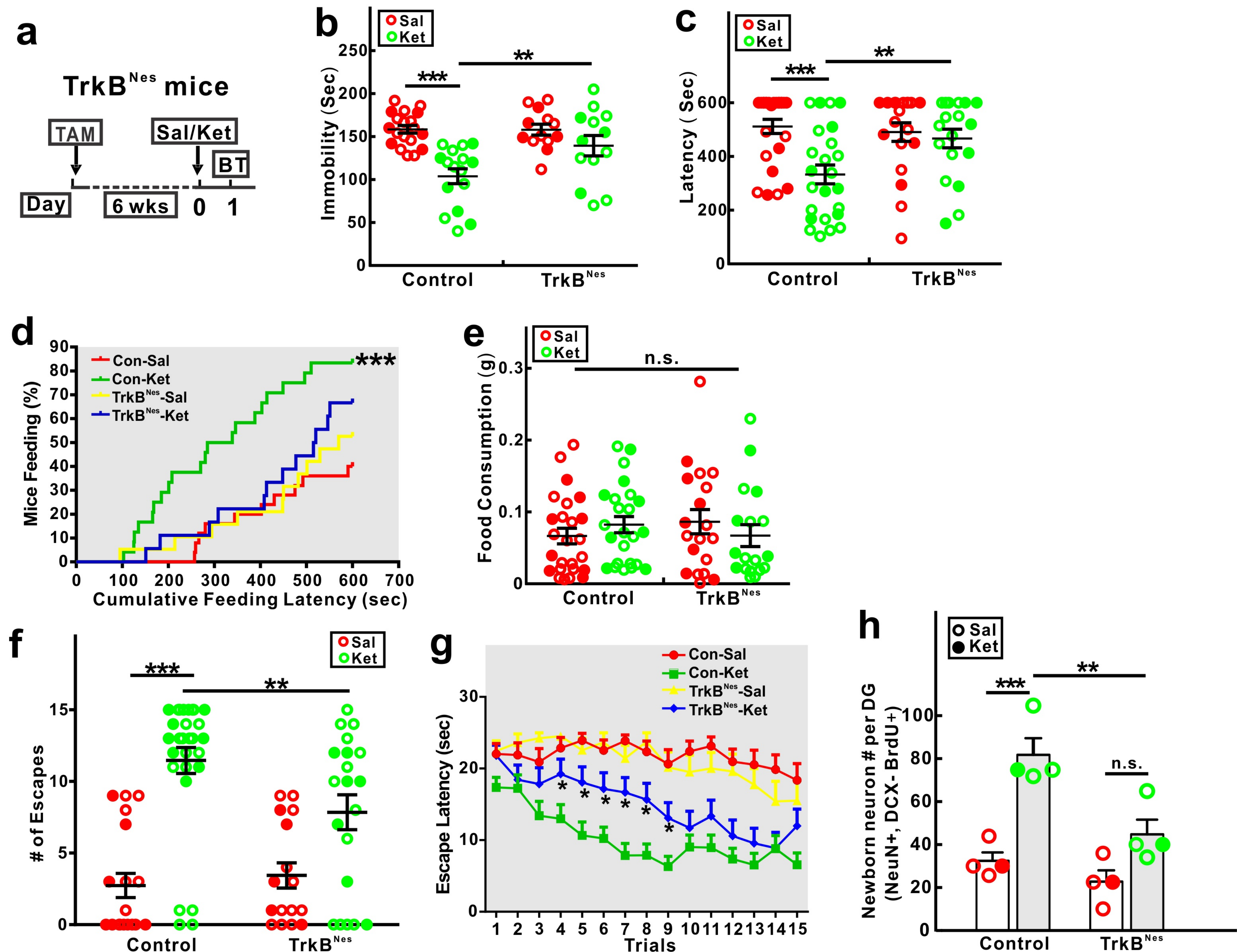

Supplementary Figure 7. The antidepressant effect of ketamine is blocked by TrkB deletion in newborn cells. (**a**) Mice were induced with tamoxifen 6 weeks before saline or ketamine treatment. Twenty four hours after saline or ketamine treatment, mice were exposed to a battery of behavior tests (BT) to evaluate the anti-depressive like effect.

(**b**) $TrkB^{Nes}$ f/f mice do not show an antidepressant response to ketamine in the forced-swim test (n = 19, 16, 13, 13 for each group, respectively and for each group, female mice represented 6, 6, 5, 4, respectively; two-way ANOVA with unweighted mean analysis, $F(1, 57) = 5.122$, $p = 0.0274$ for genotype X treatment interaction, $F(1, 57) = 4.870$, $p = 0.0314$ for genotype, $F(1, 57) = 21.211$, $p < 0.0001$ for treatment; Tukey post hoc, ***p = 2.345E-6 for saline vs ketamine in control mice; **p = 0.003 for ketamine treatment in control vs $TrkB^{Nes}$ f/f mice).

(**c**, **d, e**) In the novelty-suppressed feeding test, ketamine decreased feeding latency in control mice, but not in $TrkB^{Nes}$ f/f (c) (n = 25, 24, 19, 18 for each group, respectively and among them, female mice represented 8, 8, 7, 6 for each group, respectively; two-way ANOVA with unweighted mean analysis, $F (1, 82) = 5.533$, $p = 0.0211$ for genotype X treatment interaction, $F (1, 82) = 2.977$, $p = 0.0882$ for genotype, $F (1, 82) = 9.525$, $p = 0.0028$ for treatment; Tukey post hoc, ***p = 8.195E-5 for saline vs ketamine in control mice; **p = 0.006 for ketamine treatment in control vs $TrkB^{Nes}$ f/f mice). The cumulative feeding latency was measured in (d) (Mantel-Cox log-rank test ***p = 0.0006 for ketamine treatment in control mice compared with all other 3 groups; not significant in other comparisons). All group of mice consume the same amount of food suggesting that there are no confounding effects on appetitive behavior (e) (Two-way ANOVA with unweighted mean analysis, $F (1, 82) = 1.729$, $p = 0.1923$ for genotype X treatment interaction, $F (1, 82) = 0.03096$, $p = 0.8608$ for genotype, $F (1, 82) = 0.01615$, $p = 0.8992$ for treatment; not significant, n.s.).

(**f**, **g**) In the learned-helplessness test, ketamine treated $TrkB^{Nes}$ f/f mice escaped from the shocked chamber fewer times compared with ketamine treated control mice (f) (n = 19, 28, 16, 20 for each group, respectively and among them, female mice are 6, 8, 5, 7 for each group, respectively; Two-way ANOVA with unweighted mean analysis, $F (1, 79) = 4.540$, $p = 0.0362$ for genotype X treatment interaction, $F (1, 79) = 2.070$, $p = 0.1542$ for genotype, $F (1, 79) = 42.10$, $p < 0.0001$ for treatment; Tukey post hoc, ***p = 6.699E-

9 for saline vs ketamine in control mice; **p = 0.008 for ketamine treatment in control vs trkB^{Nes} f/f mice). Similarly, the latency to escape in trials 4 - 9 is significantly increased in ketamine treated TrkB^{Nes} f/f mice (g) (Repeated-measures ANOVA, F (3, 79) = 20.34, p < 0.0001. *p < 0.05 for ketamine treated trkB^{Nes} f/f mice vs ketamine treated control mice (p = 0.0393, 0.0088, 0.0159, 0.0010, 0.0047 and 0.0359 for trials 4-9, respectively)).

(**h**) Quantification of newborn neurons (BrdU+/NeuN+/DCX-) produced in the DG following the paradigm shown in Fig.5b (n = 4 per group; two-way ANOVA, F(1, 12) = 4.904, p = 0.0469 for genotype X treatment interaction, F(1, 12) = 14.31, p = 0.0026 for genotype, F(1, 12) = 33.42, p < 0.0001 for treatment; Tukey post hoc, ***p = 0.0005 for saline vs ketamine in control mice; **p = 0.0054 for ketamine treated control vs TrkB^{Nes} f/f mice; p = 0.1066 for saline vs ketamine in TrkB^{Nes} f/f mice, not significant- n.s.)

Circles in solid color denote the data points from female mice.

Supplementary Fig. 8

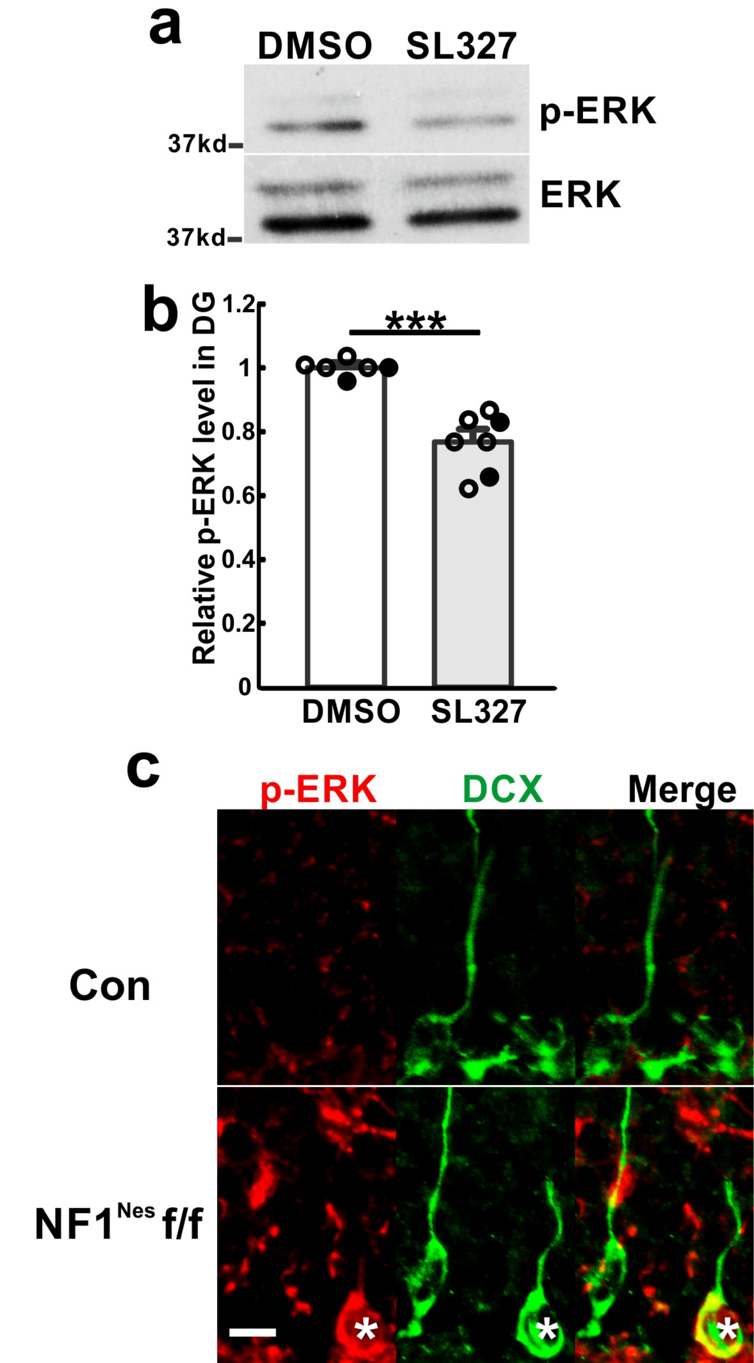

Supplementary Figure 8. ERK activation in the DG is modulated by the MEK inhibitor and NF1 cKO.

(**a**, **b**) The immunoblot analysis shows that phospho-ERK is significantly decreased by about 25% in the DG of SL327 treated mice (n = 6, 7 for DMSO and SL327, respectively; ***p = 0.0001 by unpaired two-tailed *t*-test). Circles in solid color denote the data points from female mice.

(**c**) Confocal representative images show $NF1^{Nestin}$ f/f mice display enhanced ERK pathway activity in DCX+ cells compared with littermate controls three weeks after tamoxifen induction. Asterisk denotes a representative DCX+ cell that has greatly increased the p-ERK level. Scale bar, 10 µm.

Supplementary Fig.9

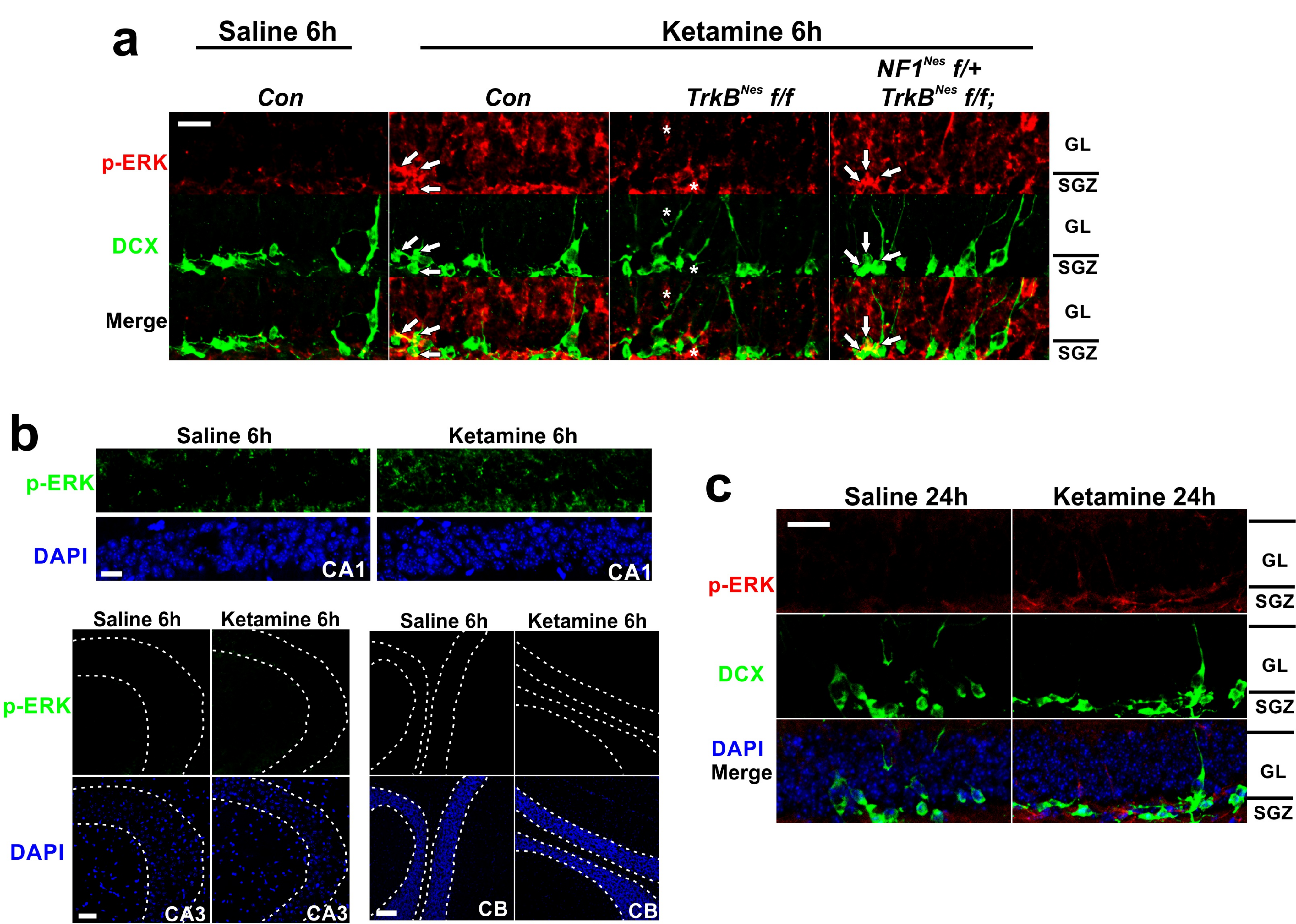

Supplementary Figure 9. NF1 ablation restores ERK activation in TrkB knockout mice. (a) Representative confocal images show greatly increased p-ERK (red) levels in DCX+ cells (green) in control mice six hours after ketamine treatment compared with saline-treated control mice. p-ERK is not increased in ketamine-treated $TrkB^{Nes}$ f/f mice. The p-ERK level in $TrkB^{Nes}$ f/f; $NF1^{Nes}$ f/+ mice is comparable to ketamine treated control mice. DCX+ cells that contain strong p-ERK signal are highlighted by arrows and only can be found in control and $TrkB^{Nes}$ f/f; $NF1^{Nes}$ f/+ double mutant mice. Some representative strong p-ERK signal (asterisks) in $TrkB^{Nes}$ f/f sections are not colocalized with DCX+ cells. Scale bar, 20 µm.
(**b**) The p-ERK level in other brain regions was examined with 6 hours of ketamine treatment. p-ERK level (green) increases in the CA1 region, but not in the CA3 (circled by dash-lines) and cerebellum (CB, granule cell layer highlighted by dash-lines). Scale bar, 20 µm, 50 µm, 100 µm for the images of CA1, CA3, and cerebellum, respectively.
(**c**) Twenty-four hours after ketamine treatment, p-ERK (red) level decreases in the granular layer (GL) of the DG, but still, display high level in DCX+ cells (green) and other progenitors in the subgranular zone (SGZ) compared with saline-treated mice. Scale bar, 20 µm.

Supplementary Fig. 10

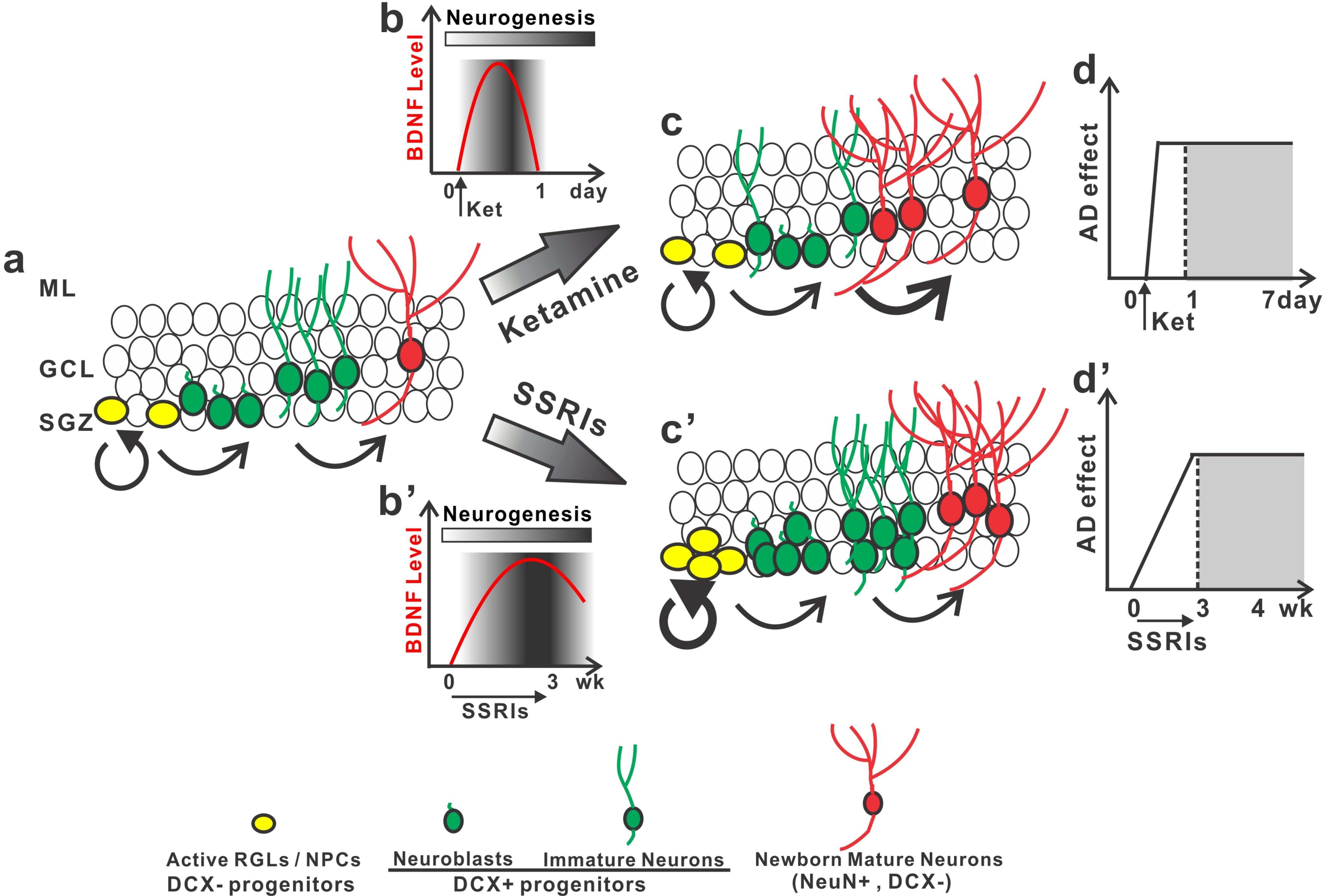

Supplementary Figure 10. A model proposed for the different role that BDNF may play in mediating adult hippocampal neurogenesis and anti-depressive response to antidepressants. (**a**) Neural progenitor cells (NPCs) that are derived from self-renewing radial-glia like stem cells (RGLs) (yellow cells), undergo rapid proliferation to produce DCX+ progenitors (green cells) including neuroblasts and immature neurons, which in turn differentiate into NeuN expressing mature neurons (red cells). (**b, c**) After one low dose of ketamine, BDNF protein levels in the DG are increased rapidly and transiently over 24 hours, resulting in accelerated DCX+ progenitor differentiation into NeuN+ neurons and reduction of the DCX+ progenitor pool. (**d**) This BDNF dependent neurogenic event is essential to sustain the anti-depressive response to ketamine beyond 24 hours (gray area). (**b', c'**) Chronic treatment of conventional SSRI antidepressants modulates BDNF levels gradually, leading to enhanced neurogenesis that is sustained for a relatively long period of time. In this scenario, DCX negative RGLs and IPCs respond to BDNF with increased production and survival of transit amplifying cells that take weeks to generate functional newborn granule neurons, (**d'**) which then produce a long-lasting antidepressive behavioral response (gray area).

Figure number	Total N (female N)<br>Left to Right in the chart	P value	Degree of Freedom<br>& F value
Fig. 3c	26, 25, 18, 16 (8, 7, 5, 4)	p = 0.0101 for interaction<br>p < 0.0001 for genotype<br>p = 0.0028 for treatment	F(1,81) = 6.938<br>F(1,81) = 19.16<br>F(1,81) = 9.527
Fig. 3d	8, 8, 5, 6 (no females)	p = 0.0141 for interaction<br>p = 0.0457 for genotype<br>p = 0.0339 for treatment	F(1,23) = 7.060<br>F(1,23) = 4.462<br>F(1,23) = 5.087
Fig. 4g	4 for each group (no females)	p < 0.0001 for interaction<br>p < 0.0001 for V/T treatment<br>p < 0.0001 for S/K treatment	F(1,12) = 103.2<br>F(1,12) = 497<br>F(1,12) = 208.9
Fig. 4h	19, 21, 16, 16 (6, 6, 4, 5)	p = 0.0032 for interaction<br>p < 0.0001 for V/T treatment<br>p = 0.0001 for S/K treatment	F(1,68) = 9.339<br>F(1,68) = 19.37<br>F(1,68) = 16.24
Fig. 5c	4 for each group (no females)	p = 0.0055 for interaction<br>p = 0.0092 for genotype<br>p < 0.0001 for treatment	F(1,12) = 11.37<br>F(1,12) = 9.618<br>F(1,12) = 37.96
Fig. 5d	4 for each group (no females)	p = 0.0160 for interaction<br>p = 0.0610 for genotype<br>p = 0.0063 for treatment	F(1,12) = 7.842<br>F(1,12) = 4.273<br>F(1,12) = 10.89
Fig. 5e	9, 10, 7, 7 (2, 3, 2, 2)	p = 0.3644 for interaction<br>p = 0.9714 for genotype<br>p < 0.0001 for treatment	F(1,29) = 0.8493<br>F(1,29) = 0.0013<br>F(1,29) = 40.58
Fig. 5f	18, 17, 15, 15 (6, 6, 5, 5)	p < 0.0001 for interaction<br>p = 0.0001 for genotype<br>p < 0.0001 for treatment	F(1,61) = 20.49<br>F(1,61) = 17.07<br>F(1,61) = 17.52
Fig. 5g	25, 23, 18, 17 (6, 6, 5, 4)	p = 0.0069 for interaction<br>p < 0.0001 for genotype<br>p < 0.0001 for treatment	F(1,79) = 7.704<br>F(1,79) = 20.31<br>F(1,79) = 28.86
Fig. 5h	14, 12, 11, 13 (3, 3, 3, 3)	p = 0.0376 for interaction<br>p = 0.0004 for genotype<br>p = 0.0006 for treatment	F(1,46) = 4.586<br>F(1,46) = 14.51<br>F(1,46) = 13.66
Fig. 5j	14, 12, 11, 13 (3, 3, 3, 3)	p = 0.6525 for interaction<br>p = 0.9650 for genotype<br>p = 0.3425 for treatment	F(1,46) = 0.2055<br>F(1,46) = 0.00194<br>F(1,46) = 0.9198
Fig. 7b	3, 3, 3, 5 (no females)	p = 0.0005 for interaction<br>p = 0.0006 for inhiitor<br>p < 0.0001 for treatment	F(1,10) = 26.02<br>F(1,10) = 23.81<br>F(1,10) = 62.77
Fig. 7c	5, 5, 10, 10 (1, 1, 3, 3)	p = 0.0014 for interaction<br>p = 0.0202 for inhibitor<br>p = 0.0043 for treatment	F(1,26) = 12.88<br>F(1,26) = 6.119<br>F(1,26) = 9.806
Fig. 7e	3, 4, 4, 5 (no females)	p = 0.0110 for interaction<br>p = 0.0300 for genotype<br>p = 0.0320 for treatment	F(1,12) = 9.012<br>F(1,12) = 6.057<br>F(1,12) = 5.883
Fig. 7f	10, 11, 7, 6, 10, 11 (3, 4, 3, 3, 4, 4)	p < 0.0001 for interaction<br>p = 0.1371 for genotype<br>p < 0.0001 for treatment	F(2,49) = 11.95<br>F(2,49) = 2.070<br>F(1,49) = 22.42
Fig. 8a	22, 19, 14, 19, 9, 10, 8, 10, 12, 12<br>(8, 7, 5, 6, 3, 3, 3, 3, 4, 4)	p = 0.0386 for interaction<br>p = 0.0136 for genotype<br>p < 0.0001 for treatment	F(4,125) = 2.611<br>F(4,125) = 3.278<br>F(1,125) = 50.82
Fig. 8b	5, 5, 4, 4, 4, 4 (no females)	p = 0.0106 for interaction<br>p < 0.0001 for genotype<br>p = 0.0001 for treatment	F(2,20) = 5.754<br>F(2,20) = 15.29<br>F(1,20) = 21.74
Fig. 8c	5, 5, 4, 4, 4, 4 (no females)	p = 0.0215 for interaction<br>p = 0.0011 for genotype<br>p = 0.0001 for treatment	F(2,20) = 4.68<br>F(2,20) = 9.812<br>F(1,20) = 23.16

Supplementary Table 1, Two-way ANOVA statistical analysis information for main figures
